# Protective Effects of Aqueous Extracts of *Flos lonicerae Japonicae* against Hydroquinone-Induced Toxicity in Hepatic L02 Cells

**DOI:** 10.1155/2018/4528581

**Published:** 2018-11-18

**Authors:** Yanfang Gao, Huanwen Tang, Liang Xiong, Lijun Zou, Wenjuan Dai, Hailong Liu, Gonghua Hu

**Affiliations:** ^1^Department of Preventive Medicine, Gannan Medical University, 1 Yixueyuan Road, Ganzhou, 341000 Jiangxi, China; ^2^Department of Environmental and Occupational Health, Dongguan Key Laboratory of Environmental Medicine, School of Public Health, Guangdong Medical University, Dongguan, 523808 Guangdong, China; ^3^Department of Occupational Health and Toxicology, School of Public Health, Nanchang University, BaYi Road 461, Nanchang, 3300061 Jiangxi, China

## Abstract

Hydroquinone (HQ) is widely used in food stuffs and is an occupational and environmental pollutant. Although the hepatotoxicity of HQ has been demonstrated both in vitro and in vivo, the prevention of HQ-induced hepatotoxicity has yet to be elucidated. In this study, we focused on the intervention effect of aqueous extracts of *Flos lonicerae Japonicae* (FLJ) on HQ-induced cytotoxicity. We demonstrated that HQ reduced cell viability in a concentration-dependent manner by administering 160 *μ*mol/L HQ for 12 h as the positive control of cytotoxicity. The aqueous FLJ extracts significantly increased cell viability and decreased LDH release, ALT, and AST in a concentration-dependent manner compared with the corresponding HQ-treated groups in hepatic L02 cells. This result indicated that aqueous FLJ extracts could protect the cytotoxicity induced by HQ. HQ increased intracellular MDA and LPO and decreased the activities of GSH, GSH-Px, and SOD in hepatic L02 cells. In addition, aqueous FLJ extracts significantly suppressed HQ-stimulated oxidative damage. Moreover, HQ promoted DNA double-strand breaks (DSBs) and the level of 8-hydroxy-2′-deoxyguanosine and apoptosis. However, aqueous FLJ extracts reversed HQ-induced DNA damage and apoptosis in a concentration-dependent manner. Overall, our results demonstrated that the toxicity of HQ was mediated by intracellular oxidative stress, which activated DNA damage and apoptosis. The findings also proved that aqueous FLJ extracts exerted protective effects against HQ-induced cytotoxicity in hepatic L02 cells.

## 1. Introduction

Hydroquinone (HQ) is a ubiquitous environmental chemical in cosmetics, medicines, the environment, and human diet; HQ can be metabolized from benzene as potentially hematotoxic, genotoxic, and carcinogenic compounds [1]. Humans are exposed to HQ through various channels, including oral administration, inhalation, and through the skin [2, 3]. Although the effects of HQ exposure on human health have been extensively studied and reported, the actual mechanisms of such effects remain unclear. The involved mechanisms trigger oxidative stress, which causes DNA damage, mutation in cellular transformation, in vivo tumorigenesis, gene toxicity, and epigenetic changes [1, 4–8]. In our previous experiments, HQ induced apoptosis in hepatic L02 cells by changing the cellular redox status by reducing the cellular thiol level and increasing the cellular reactive oxygen species (ROS) level. In addition, ROS can lead to DNA damage by breaking DNA or producing lipid peroxidation in the membrane, thereby increasing the degrees of apoptosis and necrosis of L02 hepatocytes [1, 9–11]. These findings indicated that HQ can damage L02 hepatocytes through a series of oxidative stress reactions, so it is possible to use some antioxidants for reducing HQ toxicity.

*Flos lonicerae Japonicae* (FLJ) is the flower of *Lonicera japonica Thunb*, which is widely planted in China [12]. FLJ is used worldwide as a popular traditional herbal medicine with various pharmacological activities [13, 14]. In traditional Chinese medicine, FLJ is typically used to treat common colds and fevers. FLJ exerts various effects, such as antioxidant, anti-inflammatory, antihyperlipidemic, and anticancer [15–18]. In addition, FLJ protects cells against hydrogen peroxide-induced apoptosis by phosphorylating MAPKs and PI3K/Akt. FLJ is believed to dispel noxious heat from the blood and neutralize poisonous effects. FLJ significantly increases blood neutrophil activity and promotes neutrophil phagocytosis at appropriate concentrations [12]. Some investigators posited that the methanol extract of FLJ induces protective effects against rat hepatic injuries caused by carbon tetrachloride and aqueous extracts of FLJ flowers may act as therapeutic agents for inflammatory disease through the selective regulation of NF-*κ*B activation in rat liver [19]. FLJ is characterized by high biomass, easy cultivation, extensive competitive ability, wide geographic distribution, and strong resistance to environmental stresses, including bacterial, viral, and oxidative stresses [20]. However, the protective effects of aqueous FLJ extracts against HQ-induced cytotoxicity have not been demonstrated.

In this study, we examined the protective effects of aqueous FLJ extracts against HQ-induced cytotoxicity and their involvement in oxidative stress, DNA damage, and apoptosis. For this reason, we investigated the MDA and LPO levels as indexes of lipid peroxidation; the activities of SOD, GSH, and GSH-Px as antioxidant enzymes; DNA double-strand breaks (DSBs) and 8-hydroxy-2′-deoxyguanosine (8-OHdG) level as specific markers of oxidative damage of DNA; HQ-induced apoptosis; and the cytoprotective effect of aqueous FLJ extracts in hepatic L02 cells.

## 2. Materials and Methods

### 2.1. Chemicals

HQ, 3-(4,5-dimethylthiazol-2-yl)2,5-diphenyl-tetrazolium bromide (MTT), Hoechst33258, low-melting agarose (LMA), normal-melting agarose (NMA), 8-OHdG, deoxyguanosine (dG), and propidium iodide (PI) were purchased from Sigma (St. Louis, MO, USA). Fetal bovine serum (FBS) and RPMI-1640 medium were acquired from HyClone (Logan, UT, USA). Penicillin-streptomycin for the cell culture and trypsin were procured from Gibco/Invitrogen (Carlsbad, CA, USA). Other chemicals and reagents were of the highest analytical grade and bought from Sangon Biotech Co. Ltd. (Shanghai, China).

### 2.2. Plant Material and Preparation of Extract

The aqueous FLJ extracts were prepared by adopting the standard method used for treating patients with liver disease in traditional Chinese medicine. In brief, dried FLJ fruits (100 g) were boiled in 500 mL of distilled water for 3 h. The total extract was centrifuged at 5000 rcf for 30 min. The supernatant was filtered with filter paper, and the residue was further extracted twice under the same conditions. The filtrates were evaporated to dryness under vacuum and weighed. The final yield was 12.5% (*w*/*w*). The lyophilized extract was dissolved in distilled water to produce a final concentration of 100 g/mL FLJ extract, which was stored at −20°C.

### 2.3. Cell Lines and Culture

The immortalized human normal hepatocyte L02 cell line was provided by Dr. Zhixiong Zhuang (Shenzhen Center for Disease Control and Prevention, Guangdong, China). L02 cells were cultured in RPMI-1640 medium supplemented with 10% (*v*/*v*) heat-inactivated FBS and antibiotic supplement (100 U/mL of penicillin and 100 *μ*g/mL of streptomycin) in a humidified incubator at 37°C under 95% air and 5% CO_2_. After each specified treatment time of incubation, the cells were harvested for further analysis.

### 2.4. Assessment of L02 Cell Viability

The cell viability of L02 cells was determined by MTT assay. The cells were seeded into 96-well flat-bottomed plates overnight at 37°C under 5% CO_2_ and immediately treated with HQ (5, 10, 20, 40, 80, and 160 *μ*M) for 6, 12, 24, and 48 h; 160 *μ*mol/L HQ was administered for 12 h as the positive control of oxidative damage. The samples were classified under five groups, namely, control (without HQ and FLJ), 160 *μ*mol/L HQ, 0.25 g/mL FLJ + HQ, 0.50 g/mL FLJ + HQ, and 1.00 g/mL FLJ + HQ for 12 h. Subsequently, 20 *μ*L of MTT solution (5 mg/mL) was added to the culture medium for 4 h before the end of the treatment time to allow the formation of formazan crystals. The supernatant was discarded, and 100 *μ*L of cell lysis buffer (50% DMF, 20% SDS, pH 4.6–4.7) was added to dissolve the intracellular crystalline formazan products. After constant and gentle shaking for 10 min at room temperature, the absorbance was recorded at 570 nm. Cell viability was calculated using the equation cellular relative viability = (OD_treated wells_ − OD_blank_)/(OD_control wells_ − OD_blank_).

### 2.5. Measurement of Intracellular Cytotoxicity and Oxidative Damage

The toxic effect of HQ in hepatic L02 cells was estimated in terms of LDH release and the activities of ALT and AST by using commercial kits (Nanjing Jiancheng Bioengineering Institute, China) in accordance with the manufacturer's protocol.

Commercial kits (Nanjing Jiancheng Bioengineering Institute, China) were utilized to assess the activities of SOD, GSH-Px, and GSH and the levels of MDA and LPO. The activities of SOD, GSH-Px, and GSH were expressed as units/milligram of protein. MDA and LPO contents were expressed as nmol/milligram of protein. The protein concentration was estimated using a BCA kit.

### 2.6. DNA Damage Assay

DNA damage was determined by the following assays. (i) DNA damage was evaluated using alkaline single-cell gel electrophoresis (comet assay). In brief, after 24 h of exposure, followed by washing with PBS, a 1.2 × 10^5^ cell suspension was mixed with 0.8% LMA at 37°C and spread on a fully frosted microscope slide precoated with 0.65% NMA. After the agarose solidified, the slide was covered with another 75 *μ*L of 0.8% LMA and then immersed in lysis solution (2.5 M NaCl, 100 mM Na_2_EDTA, 10 mM Tris base, 1% Triton-X 100, 10% DMSO, pH 10) for 2 h at 4°C. The slides were placed in a gel-electrophoresis apparatus containing 300 mM NaOH and 1 mM Na_2_EDTA (pH 13) for 30 min at 4°C to allow DNA unwinding and alkali labile damage. Electrophoresis was performed at 25 V (300 mA) and 4°C for 20 min. Subsequently, all slides were washed three times with a neutralizing buffer (0.4 M Tris, pH 7.5) for 5 min each time and stained with 80 *μ*L of PI (5 *μ*g/mL). A total of 100 randomly chosen cells (comets) were visually scored using a fluorescence microscope (Nikon Eclips TE-S, Japan) equipped with an excitation filter of 515–560 nm and a barrier filter of 590 nm. The “tail length and tail moment” of each comet were calculated using casp-1.2.2 analysis software. (ii) 8-OHdG was evaluated by high-performance liquid chromatography with electrochemical detection (HPLC-ECD) [21]. In brief, genomic DNA was extracted using a Genomic DNA Purification Kit in 500 *μ*g hydrolyzed DNA samples through the nuclease P1 and alkaline phosphatase hydrolysis of DNA. The samples were then filtered through 0.22 *μ*m nylon filters. 8-OHdG and dG levels were measured by using HPLC-ECD and HPLC with variable wavelength detector (HPLC-UV) systems as previously described. About 100 *μ*L of final hydrolysates was analyzed by HPLC-ECD with reverse phase-C18 (RP-C18) analytical column as the column. The mobile phase consisted of 50 mM KH_2_PO_4_ buffer (pH 5.5 and containing 10% methanol). The separations were performed at a flow rate of 1 mL/min. The amount of 8-OHdG in DNA was calculated as the number of 8-OHdG molecules/10^6^ unmodified dG molecules.

### 2.7. Apoptosis Assay

Apoptosis was assessed by flow cytometry using PI assay. In brief, the cells were incubated in RPMI 1640 with 10% FBS. The cells consisted of the control group, the group treated with 160 *μ*M HQ, and the group coincubated in the absence or presence of FLJ (0.25, 0.50, and 1.00 g/mL) for 12 h. Subsequently, the cells were washed with cold PBS and incubated with PI (0.5 *μ*g) for 20 min at room temperature in the dark. The cells were immediately analyzed on a FACSCanto II flow cytometer (Becton and Dickinson, San Jose, CA, USA), and data from 10,000 events were obtained. The results were expressed as the percentage of cells labeled with PI (apoptotic).

### 2.8. Statistical Analysis

All data are presented as the mean ± standard deviation (SD). Statistical evaluation of data analysis was performed using SPSS 16.0 for Windows. The differences between the mean values of multiple groups were analyzed by one-way ANOVA, followed by SNK test. *P* < 0.05 was considered statistically significant.

## 3. Results

### 3.1. Protective Effects of Aqueous FLJ Extracts against HQ-Induced Cytotoxicity

**Figure 1:**
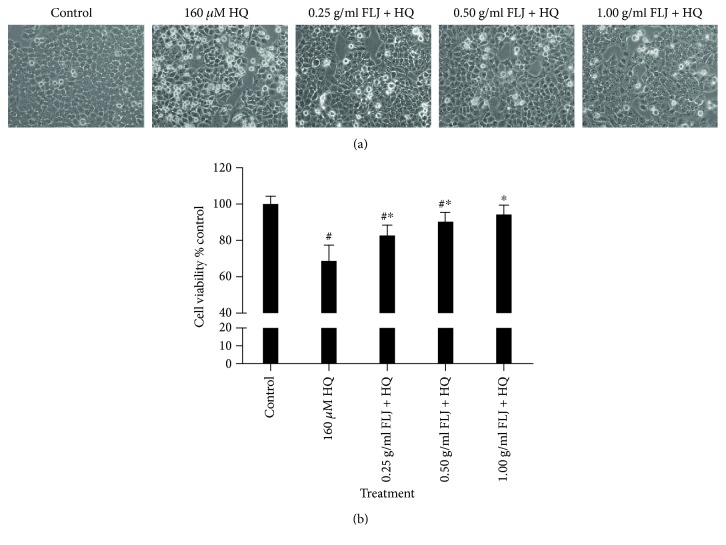
Protective effects of aqueous FLJ extracts against HQ-induced cytotoxicity (a) L02 cells treated with 0 and 160 *μ*M HQ and 0.25, 0.50, and 1.00 g/mL FLJ + HQ for 12 h. Morphological features of L02 cells were observed by inverted microscope. (b) L02 cells treated with 0 and 160 *μ*M HQ and 0.25, 0.50, and 1.00 g/mL FLJ + HQ for 12 h. Cell proliferation was detected by using the MTT proliferating reagent. All data were representative of at least three independent experiments. ^#^*P* < 0.05 compared with the control. ^∗^*P* < 0.05 compared with 160 *μ*M HQ treatment.

As the first step in determining the necessary HQ concentration to induce cytotoxicity, the viability of L02 cells was assessed by MTT assay. HQ induced a concentration- and time-dependent reduction in L02 cell viability compared with untreated control cells. We administered 160 *μ*mol/L HQ for 12 h as the positive control of cytotoxicity. After 12 h of coexposure to aqueous FLJ extracts at the specified concentrations, the cell significantly increased compared with that in the corresponding HQ-treated groups (Figures 1(a) and 1(b)). These results revealed that aqueous FLJ extracts intervened the HQ-induced cytotoxicity.

**Figure 2:**
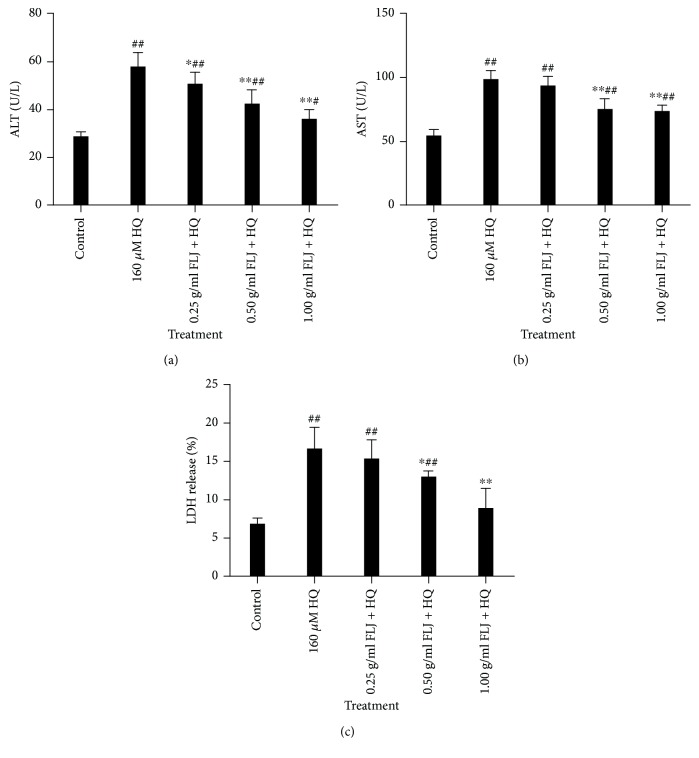
Protective effects of aqueous FLJ extracts against HQ-induced cytotoxicity L02 cells treated with 0 and 160 *μ*M HQ and 0.25, 0.50, and 1.00 g/mL FLJ + HQ for 12 h. LDH release and the activities of aminotransferase (ALT) and aspartate aminotransferase (AST) were detected by using commercial kits. All data were representative of at least three independent experiments. ^#^*P* < 0.05 compared with the control. ^∗^*P* < 0.05 compared with 160 *μ*M HQ treatment.

To evaluate the protective effects of aqueous FLJ extracts against HQ, we examined cytotoxicity in terms of the LDH release and the ALT and AST levels. The results showed that aqueous FLJ extracts significantly downregulated LDH release, ALT, and AST in a concentration-dependent manner compared with the corresponding HQ-treated groups (Figure 2). Thus, aqueous FLJ extracts could reverse HQ-induced cytotoxicity.

### 3.2. Protective Effects of Aqueous FLJ Extracts against HQ-Induced Cellular Oxidative Damage

**Figure 3:**
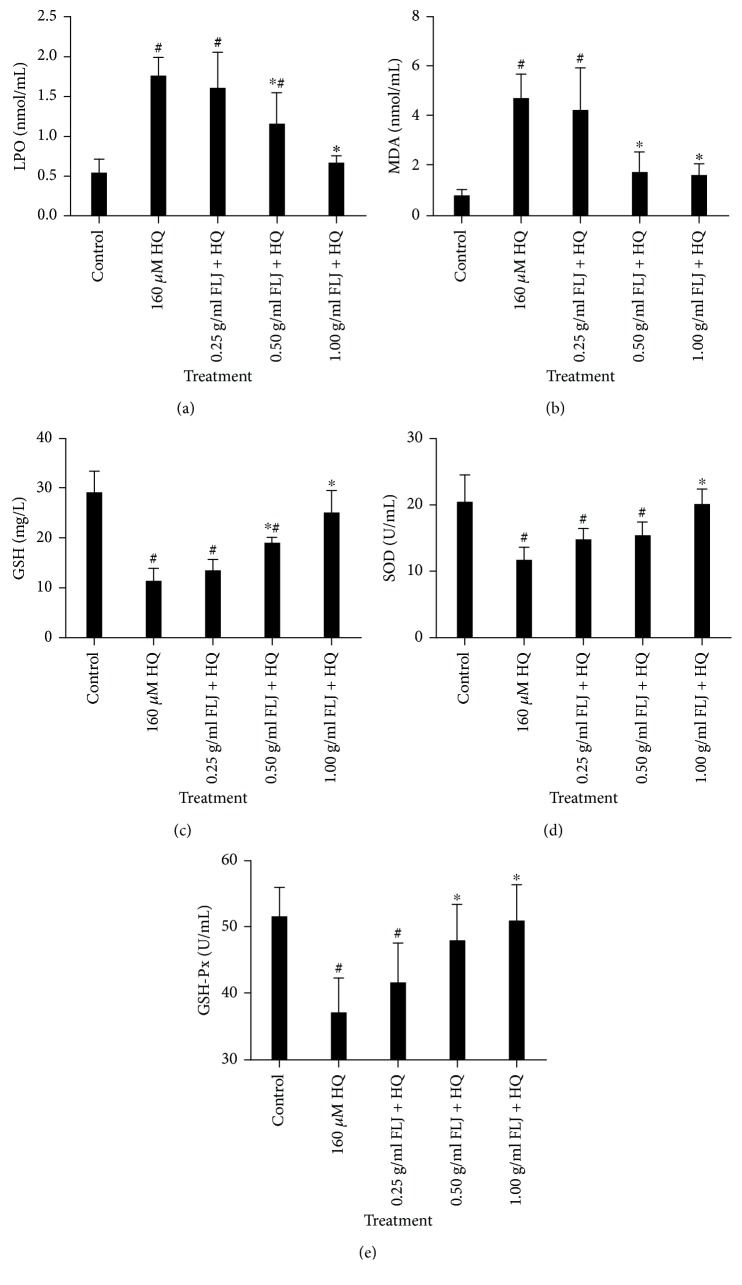
Protective effects of aqueous FLJ extracts against HQ-induced oxidative damage L02 cells treated with 0 and 160 *μ*M HQ and 0.25, 0.50, and 1.00 g/mL FLJ + HQ for 12 h. MDA and LPO levels as the indexes of lipid peroxidation and the SOD, GSH, and GSH-Px activities as antioxidant enzymes were detected by using commercial kits for oxidative stress. All data were representative of at least three independent experiments. ^#^*P* < 0.05 compared with the control. ^∗^*P* < 0.05 compared with 160 *μ*M HQ treatment.

Oxidative stress can mediate apoptosis in various cell models and is considered an important apoptotic signal. Therefore, we investigated whether intracellular oxidative damage is involved in HQ-induced cell death and found that HQ exposure increased MDA and LPO production, but aqueous FLJ extracts reduced the production of MDA and LPO in a concentration-dependent manner. In addition, aqueous FLJ extracts reversed the production of antioxidant enzymes (such as GSH, SOD, and GSH-Px), indicating that aqueous FLJ extracts exerted a protective effect against HQ-induced oxidative stress in L02 cells (Figure 3).

### 3.3. Protective Effects of Aqueous FLJ Extracts against HQ-Induced DNA Damage

**Figure 4:**
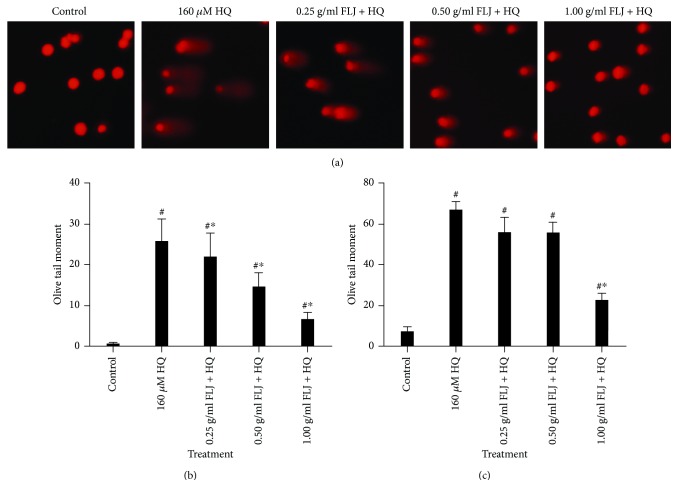
Protective effects of aqueous FLJ extracts against HQ-induced DNA damage (a, b) L02 cells treated with 0 and 160 *μ*M HQ and 0.25, 0.50, and 1.00 g/mL FLJ + HQ for 12 h. DNA damage was evaluated by alkaline single-cell gel electrophoresis (comet assay). (c) L02 cells treated with 0 and 160 *μ*M HQ and 0.25, 0.50, and 1.00 g/mL FLJ + HQ for 12 h. 8-Hydroxy-2′-deoxyguanosine (8-OHdG) level as a specific marker of oxidative damage of DNA. 8-OHdG was evaluated using high-performance liquid chromatography with electrochemical detection (HPLC-ECD). All data were representative of at least three independent experiments. ^#^*P* < 0.05 compared with the control. ^∗^*P* < 0.05 compared with 160 *μ*M HQ treatment.

To elucidate the protective effects of aqueous FLJ extracts against HQ-induced DNA damage, L02 cells were exposed to various treatments: 0 and 160 *μ*M HQ and 0.25, 0.5, and 1.0 g/mL FLJ + HQ for 12 h. DNA damage was assessed. We performed comet assay, which is a commonly used indicator of genomic instability and genotoxic exposure. As shown in Figure 4(a), HQ induced a marked increase in DNA damage. However, aqueous FLJ extracts significantly decreased DNA damage compared with the corresponding HQ-treated groups as measured in terms of the tail moment and tail length. To further validate our findings regarding the protective effects of aqueous FLJ extracts against HQ-induced DNA damage, we used HLPC-ECD to measure the level of 8-OHdG, which is a widely used marker of oxidative DNA damage. The results revealed a concentration-dependent decrease in 8-OHdG levels in the samples treated with aqueous FLJ extracts (Figure 4(b)), implying that aqueous FLJ extracts exerted a protective effect against HQ-induced DNA damage.

### 3.4. Protective Effects of Aqueous FLJ Extracts against HQ-Induced Apoptosis

**Figure 5:**
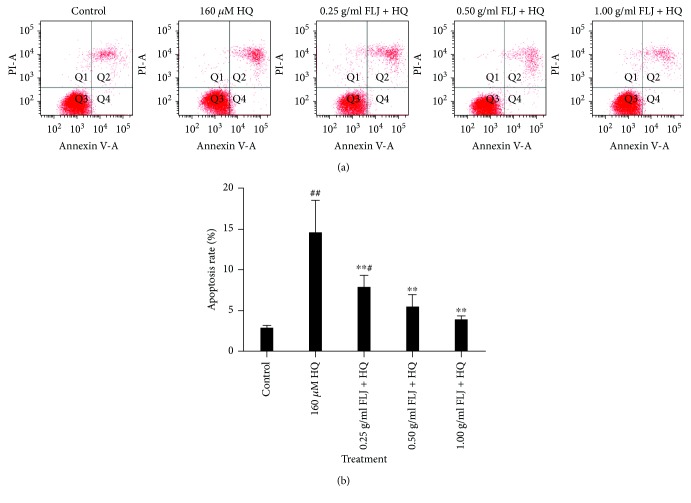
Protective effects of aqueous FLJ extracts against HQ-induced apoptosis L02 cells treated with 0 and 160 *μ*M HQ and 0.25, 0.50, and 1.00 g/mL FLJ + HQ for 12 h. Apoptosis was assessed by flow cytometry using propidium iodide (PI) assay. All data were representative of at least three independent experiments. ^#^*P* < 0.05 compared with the control. ^∗^*P* < 0.05 compared with 160 *μ*M HQ treatment.

Given that apoptosis is regulated by oxidative stress and DNA damage, the apoptosis rate in the L02 cells was also investigated by flow cytometry using PI assay. The apoptosis rate significantly decreased in the HQ-intoxicated L02 cells compared with that in the control. To further evaluate whether the protective effect of aqueous FLJ extracts on the L02 cells involves cell apoptosis, the L02 cells were incubated with aqueous FLJ extracts and HQ, and the apoptosis rate was then assessed. The results revealed that the apoptosis rate of the group exposed to both aqueous FLJ extracts and HQ was lower than that of the HQ group, indicating that aqueous FLJ extracts played an important role in reducing apoptosis in HQ-exposed L02 cells (Figure 5).

## 4. Discussion

HQ is a well-known toxicant of liver and induces many effects on the hepatic system. Although many studies have explored HQ, the actual mechanisms underlying these effects remain poorly understood. FLJ, which is also known as *Jinyinhua* or *Japanese honeysuckle*, is the dried flower bud or open flower of *Lonicera japonica Thunb* [22]. It is one of the most popular traditional Chinese medicines, and it has been applied in the healthcare of China and other East Asian countries for a long time. It has been proven to display antioxidant, antiviral, anticarcinogenic, anti-inflammatory, analgesic, antipyretic, and antimicrobial functions [23]. This study focused on the protective effects of aqueous FLJ extracts against HQ-induced cytotoxicity.

Serum ALT and AST levels are used as biochemical markers of liver damage, as the membrane destruction of hepatocytes releases hepatic enzymes, such as ALT and AST, into blood circulation [24]. The water extracts of FLJ containing 20% chlorogenic acid are protective against alcohol-induced chemical liver injury in mice [12, 24], which was similar to the protection conferred by aqueous FLJ extracts to the L02 hepatic cell model induced by HQ.

Oxidative stress plays a critical role in the development of drug-induced liver damage [25] and in HQ-induced toxicity. Intercellular ROS may cause detrimental alterations in cell membranes, DNA, and other cellular structures. ROS are critical intermediates under normal physiological conditions that contribute to pathophysiological events in liver injury. Intracellular oxidative stress balance is crucial in maintaining normal cellular function in response to exogenous and endogenous factors [26]. MDA is a key marker of lipid peroxidation. The progression of liver damage was correlated with oxidative stress, as confirmed by MDA measurements [27]. FLJ polyphenol extracts were protective against the lipid peroxidation of erythrocyte and lipid membranes [28]. Consistent with the literature, the levels of MDA and LPO in our study were significantly higher in the HQ-treated group than in the controls. The levels of MDA and LPO concentrations were lower in aqueous FLJ extracts than in the HQ group.

The primary antioxidant defense systems include SOD and the GSH redox cycle [29]. SOD, which is produced from the mitochondrion electron transfer chain and scavenges superoxide anions, is an important antioxidant enzyme. SOD can transform superoxide anions into H_2_O_2_ and catalase and then continuously detoxify them to H_2_O [30]. The GSH redox cycle, which mainly includes GSH and GPx, modulates the redox-mediated responses of hepatic cells induced by external or intracellular stimulation. GSH is the main nonenzymatic regulator of intracellular redox homeostasis. GSH directly scavenges hydroxyl radicals and is a cofactor in detoxifying hydrogen peroxide, lipid peroxides, and alkyl peroxides. GPx, which is a selenocysteine-containing enzyme, reduces lipid hydroperoxides to their corresponding alcohols and hydrogen peroxide to water in the liver. Therefore, enhancing the hepatic antioxidant system capacity may be an effective therapeutic strategy for alleviating and treating liver damage [25]. Our study showed that the cytotoxicity of HQ is mediated by intracellular oxidative stress and confirmed the protective effects exerted by aqueous FLJ extracts as an antioxidant against HQ-induced cytotoxicity in L02 cells.

DNA damage is caused by multiple factors, including oxidative stress, vitamin B12 deficiency, and ischemia-reperfusion injury [31]. The ratio of 8-OHdG/dG is correlated with the severity of oxidative stress. The high activity of an antioxidant enzyme may be a compensatory regulation in response to increased oxidative stress. In our previous study, we demonstrated that DNA damage is related to HQ-induced hepatotoxicity [11]. ROS causes oxidative stress, which leads to DNA damage. Cells have evolved elaborate mechanisms to respond to DNA damage, at the core of which is the signaling pathway known as the DNA damage checkpoint [32]. This pathway initiates many aspects of the DNA damage response (DDR), including activation of DNA repair and induction of apoptosis [33, 34]. DNA damage is an early event in DDR. Thus, we examined DNA damage through the comet assay and 8-OHdG level, which is a specific marker of oxidative damage of DNA. In different kinds of cells, HQ induces the production of superoxides and hydroperoxides, which are implicated in the initiation and promotion stages of apoptosis and DNA damage. In the present study, although HQ induced DNA damage and apoptosis, we observed a concentration-dependent reverse in 8-OHdG levels, DDBs in the nucleus, and apoptosis in the group treated with aqueous FLJ extracts.

In conclusion, the results of this study strongly suggested that aqueous FLJ extracts played a role in protecting against HQ-induced cytotoxicity. On the basis of our results, we suggest a possible mechanism involved in the protective effects of aqueous FLJ extracts against HQ-induced cytotoxicity. HQ induced MDA and LPO formation, which activated antioxidant enzyme production. Intracellular oxidative stress balance was disturbed, thereby inducing DNA damage and apoptosis and promoting HQ-induced cytotoxicity. However, aqueous FLJ extracts could increase the activation of the antioxidant, which may reduce DNA damage and apoptosis and exert a protective effect against HQ-induced cytotoxicity. The molecular mechanism could further elucidate the antioxidant role of aqueous FLJ extracts.

## Data Availability

The data used to support the findings of this study are included within the article.

## Abbreviations

DDR: DNA-damage response
HQ: Hydroquinone
MDA: Malondialdehyde
LPO: Lipid peroxidation
ROS: Reactive oxygen species
FLJ: Flos lonicerae Japonicae
LDH: Lactic dehydrogenase
ALT: Alanine transaminase
AST: Aspartate aminotransferase
PI: Propidium iodide
HPLC-EC: High-performance liquid chromatography with electrochemical
DSBs: DNA double-strand breaks
8-OHdG: 8-hydroxy-2′-deoxyguanosine.
